# Facile fabrication of screen-printed MoS_2_ electrodes for electrochemical sensing of dopamine

**DOI:** 10.1038/s41598-022-16187-2

**Published:** 2022-07-13

**Authors:** Michaela Pavličková, Lenka Lorencová, Michal Hatala, Miroslav Kováč, Ján Tkáč, Pavol Gemeiner

**Affiliations:** 1 Department of Graphic Arts Technology and Applied Photochemistry, Faculty of Chemical and Food Technology, Slovak University of Technology in Bratislava, Radlinského 9, 812 37 Bratislava, Slovak Republic; 2 Institute of Chemistry, Slovak Academy of Sciences, Dúbravská cesta 9, 845 38 Bratislava, Slovak Republic

**Keywords:** Materials science, Materials for devices

## Abstract

Molybdenum disulfide (MoS_2_) screen-printed working electrodes were developed for dopamine (DA) electrochemical sensing. MoS_2_ working electrodes were prepared from high viscosity screen-printable inks containing various concentrations and sizes of MoS_2_ particles and ethylcellulose binder. Rheological properties of MoS_2_ inks and their suitability for screen-printing were analyzed by viscosity curve, screen-printing simulation and oscillatory modulus. MoS_2_ inks were screen-printed onto conductive FTO (Fluorine-doped Tin Oxide) substrates. Optical microscopy and scanning electron microscopy with energy-dispersive X-ray spectroscopy (SEM/EDX) analysis were used to characterize the homogeneity, topography and thickness of the screen-printed MoS_2_ electrodes. The electrochemical performance was assessed through differential pulse voltammetry. Results showed an extensive linear detection of dopamine from 1 µM to 300 µM (*R*^2^ = 0.996, sensitivity of 5.00 × 10^–8^ A μM^−1^), with the best limit of detection being 246 nM. This work demonstrated the possibility of simple, low-cost and rapid preparation of high viscosity MoS_2_ ink and their use to produce screen-printed FTO/MoS_2_ electrodes for dopamine detection.

## Introduction

Two-dimensional (2D) nanomaterials have received attention in the field of chemistry, material science, physics, and nanotechnology after the successful exfoliation of graphene from graphite using Scotch tape in 2004 by Novoselov et al.^1,2^. 2D nanomaterials are defined as a class of freestanding sheet-like nanomaterials. They all have a thickness of merely a single or few atomic layers^3,4^. There has been a great effort to develop various 2D nanomaterials in recent years because of their unique properties, such as a large surface area, high chemical stability, high conductivity, mechanical strength, and optical transparency^5,6^. These materials include transition metal carbides, nitrides and carbonitrides (MXenes), hexagonal boron nitride (h-BN), graphene oxide (GO), transition metal dichalcogenides (TMDs) and many more^3,6^.

Molybdenum disulfide (MoS_2_) belongs to a class of materials called transition metal dichalcogenides (TMDs)^7^. It has a 2D layered structure where layers of Mo atoms are positioned between layers of S atoms. A strong covalent bond holds Mo-S layers while weak Van der Waals interaction exists between S layers^8,9^. MoS_2_, because of its excellent properties, such as flexibility, photoluminescence, direct bandgap (~ 1.9 eV), excellent catalytic activity and biocompatibility, can be used for applications in catalysis, transistors, energy storage and sensor devices^9–11^. Moreover, MoS_2_ has been successfully used to detect small molecules, *e*.*g*. hydrogen peroxide, glucose, eugenol, ascorbic acid, uric acid and dopamine^7,10,12^.

Dopamine (DA) is a hormone and neurotransmitter regulating the function of human metabolism, immune, hormonal, central nervous and cardiovascular systems in the human brain^10,13^. The deficiency of DA can lead to various neurological diseases, *e.g*. schizophrenia, Alzheimer's and Parkinson's disease. Hence, determining the DA concentrations is beneficial for disease diagnosis^7,14^.

Standard sensors for detecting various analytes are commonly prepared by modifying commercial screen-printed carbon electrodes (SPCE). This step is usually performed by various coating techniques^7,15,16^, especially the drop-casting method^7,10,14,17^. Drop-casting is a convenient, facile technique that requires simpler and less expensive equipment, and the applied inks do not have prescribed physical and rheological properties and chemical formulation^18,19^. However, it has several disadvantages, *e*.*g*. low reproducibility and uncontrollable distribution of the deposited material^18^. Low-viscous dispersions used for the drop-casting method have to contain suitable solvents, *e*.*g*. dimethylformamide (DMF)^7^, deionized water^20^ or *N*-metylpyrolidon (NMP)^21^. Ultrasonication and centrifugation are used for dispergation and to eliminate or reduce the unexfoliated flakes ^22^. In contrast, printing techniques (screen-printing) have high reproducibility and the possibility to prepare various fine structures for a wide range of layer thicknesses^18^. Also, the inks can be printed onto rigid or flexible substrates, and it is a low-cost, simple manufacturing process used for diverse applications^23^.

MoS_2_ sensors for dopamine detection prepared by modifying commercial screen-printed carbon electrode (SPCE) or glassy carbon electrode (GCE) have shown a low limit of detection (LOD)^7,17^. Zribi et al. modified SPCE by MoS_2_ dispersion. The MoS_2_ dispersion was prepared by ultrasonication of MoS_2_ powder in a sodium cholate water solution. They used the drop-casting method for modification and achieved LOD of 0.085 µM^17^. Moreover, many researchers combine MoS_2_ inks with graphitic materials, *e*.*g*. graphene^14^, graphitic carbon nitride^13^, graphene oxide (GO)^13,15^ or metals, such as Ag^10,16^. Importantly, these materials have good conductivity and can detect dopamine at low concentrations^16^. Cheng et al. prepared a highly sensitive electrochemical sensor with LOD of 0.007 µM constructed by modifying GCE by graphene-MoS_2_ inks^14^. Sookhakian et al. prepared Ag@MoS_2_-modified GCE with excellent selectivity, high sensitivity and a low LOD of 0.2 µM^10^. In all previously mentioned cases, the GCE was modified by the drop-casting method.

Rowley-Neale et al. prepared screen-printable carbon/MoS_2_ inks as a mixture of commercially available carbon ink and MoS_2_ powder. Their screen-printed carbon/MoS_2_ working electrodes were successfully used for electrochemical investigation of oxygen reduction reaction (ORR)^18^.

Herein, screen-printable and highly viscous MoS_2_ inks were prepared by mixing MoS_2_ with various particle sizes and an ethylcellulose binder. Inks were screen-printed onto conductive FTO (Fluorine-doped Tin Oxide) substrates. The suitability of prepared MoS_2_ inks for screen-printing was evaluated by rheological behavior analysis. The homogeneity, topography and thickness of layers were analyzed by optical microscopy and scanning electron microscopy with energy-dispersive X-ray spectroscopy (SEM/EDX) analysis. Finally, the functionality of the electrodes was studied by electrochemical measurements. This work aims at the fast and simple preparation of printable high viscosity MoS_2_ inks, which can be used to fabricate screen-printed FTO/MoS_2_ sensors for dopamine detection at a submicromolar level. To our best knowledge, this is the first screen-printed sensor based on MoS_2_ nanomaterial for the detection of dopamine.

## Experimental section

### Materials

Molybdenum disulfide (MoS_2_) powder with particle size of ~ 6 µm (max. 40 µm), molybdenum disulfide (MoS_2_) nanopowder with particle size of 90 nm, ethylcellulose (EC) (viscosity 22 cP 5% in toluene/ethanol 80:20), terpineol (≥ 96%, bp 213 °C) and FTO (Fluorine-doped Tin Oxide, 7 Ω/sq) glass substrates were purchased from Sigma Aldrich. Dopamine hydrochloride (DA, C_8_H_11_NO_2_.HCl) and phosphate buffer (PB) components (KH_2_PO_4_ and K_2_HPO_4_, pH 7.0) used for electrochemical analysis were also ordered from Sigma Aldrich. All solutions were freshly prepared in ultrapure deionized water (0.055 µS cm^−1^).

### MoS_2_ inks preparation

First, the polymeric binder was prepared by dissolving 8 wt% (EC) in terpineol. After that, MoS_2_/EC inks were prepared by dispersing 25, 45 and 60 wt% of MoS_2_ powder with particle size of ~ 6 µm (max. 40 µm) in a polymeric binder (samples Mo6-25; Mo6-45 and Mo6-60). In the case of MoS_2_ nanopowder with particle size of 90 nm, only inks with 25 and 45 wt% of MoS_2_ (samples Mo90-25 and Mo90-45) were prepared. Inks with 90 nm particle content over 45 wt% were not miscible. All inks were homogenized in a "homemade" hand-held mixing unit 5 times for 30 s.

### Screen-printing process

The MoS_2_ inks were screen-printed onto glass substrates covered by a conductive FTO layer. Before printing, FTO substrates were gradually pretreated in four steps; cleaning in detergent solution (1); acetone (2); isopropyl alcohol (3) in an ultrasonic bath, followed by UV-C treatment (4) for 20 min. For the screen-printing of prepared MoS_2_ inks, yellow high modulus polyester yarn mesh was used, with mesh count 71 cm^−1^ and 48 µm thread diameter (PME 71–48 Y, SEFAR) in combination with the stencil prepared by the photochemical way. Screen-printed MoS_2_ layers were prepared using a manual, auxiliary guide arm (constant pressure) equipped screen-printing machine (Screen Printing Table P65-80 KN, Drucktech). The printed area was 6 × 6 mm^2^. After printing, MoS_2_ layers were left for 10 min at laboratory temperature for levelling and then were dried in a laboratory oven at 120 °C for 30 min.

### Characterization methods

The rheological properties of prepared MoS_2_ inks were investigated using a rheometer (HAAKE, MARS iQ) equipped with parallel plate geometry with a diameter of 35 mm and a gap height of 0.4 mm. The temperature was set to 25 °C during all measurements. The steady-state rheological test was performed at shear rates [$\dot{\gamma }$(s^-1^)] of 0.001–1000 s^−1^ to measure the dynamic viscosity [*η* (Pa.s)] and was obtained by controlling shear rates (CR). The time-dependent controlled-shear-rate tests were performed to simulate the screen-printing process with constant shear rates in three intervals: (1) 0.5 s^−1^ for 90 s; (2) 1,000 s^−1^ for 30 s and (3) 0.5 s^−1^ for 180 s. The oscillatory tests are helpful to understand the structural changes of the inks occurring in the screen-printing process. In the shear strain amplitude sweep, the applied shear strain ranged from 0.1 to 100% at a frequency of 1 Hz, which helped to characterize the viscoelastic behavior of the inks and determine the linear viscoelastic region (LVR). Elastic and storage moduli were measured as a function of shear strain/stress. The elastic or storage modulus (${G}^{\prime}$) is related to the ability of ink to store energy and represents the elastic portion of the viscoelastic behavior. The viscous or loss modulus (${G}^{\prime\prime}$) indicates the fluidity of ink^24^. The factor tanδ, which is ${G}^{\prime\prime}/{G}^{\prime}$ gives an indication of material internal strength and helps to define viscoelasticity (Eq. (1))^25^.

1 $$tan\delta = {G}^{\prime\prime}/{G}^{\prime}$$

The thicknesses of screen-printed MoS_2_ layers were analyzed by optical microscope (LEICA DM 2700 M) using the 3D image sequential recording method. The software (Leica Application Suite V4, LEICA) was used to evaluate the arithmetic average of thickness. The adhesion of printed MoS_2_ layers to FTO substrates was tested by peel adhesion test using the adhesive tape (Scotch Crystal, 3M). Disturbance of the screen-printed layers after peeling off the adhesive tape was evaluated. The layers were backlit from below, and the images were made by optical microscopy. The topography and homogeneity of FTO/MoS_2_ layers were characterized by scanning electron microscopy with energy-dispersive X-ray spectroscopy (SEM/EDX; JEOL JSM-IT500HR). The electron acceleration voltage was set to 20 kV.

All procedures for evaluation of the performance of FTO/MoS_2_ electrodes for electrochemical sensing were run with a laboratory potentiostat/galvanostat Autolab PGSTAT 302 N with an impedimetric module (Ecochemie, Utrecht, Netherlands) in combination with NOVA 1.10 software. Differential pulse voltammetry (DPV) was used for the detection of dopamine. MoS_2_ layers onto FTO substrates were used as working electrodes, the platinum wire was used as the counter-electrode and an argentochloride electrode (Ag/AgCl/3 M KCl) as the reference electrode. Five scans were run in a plain phosphate buffer pH 7.0 in a potential window of 0–1 V to stabilize FTO/MoS_2_ electrodes. The parameters applied for the differential pulse voltammetry were as follows: 50 ms modulation time, 0.5 s interval time, 25 mV modulation amplitude, and 5 mV step. Measurements were run under Nova Software 1.10, and data acquired were evaluated using OriginPro 9.1. The limit of detection (LOD) was calculated as a signal-to-noise ratio (S/N) = 3.

## Result and discussion

**Figure 1:**
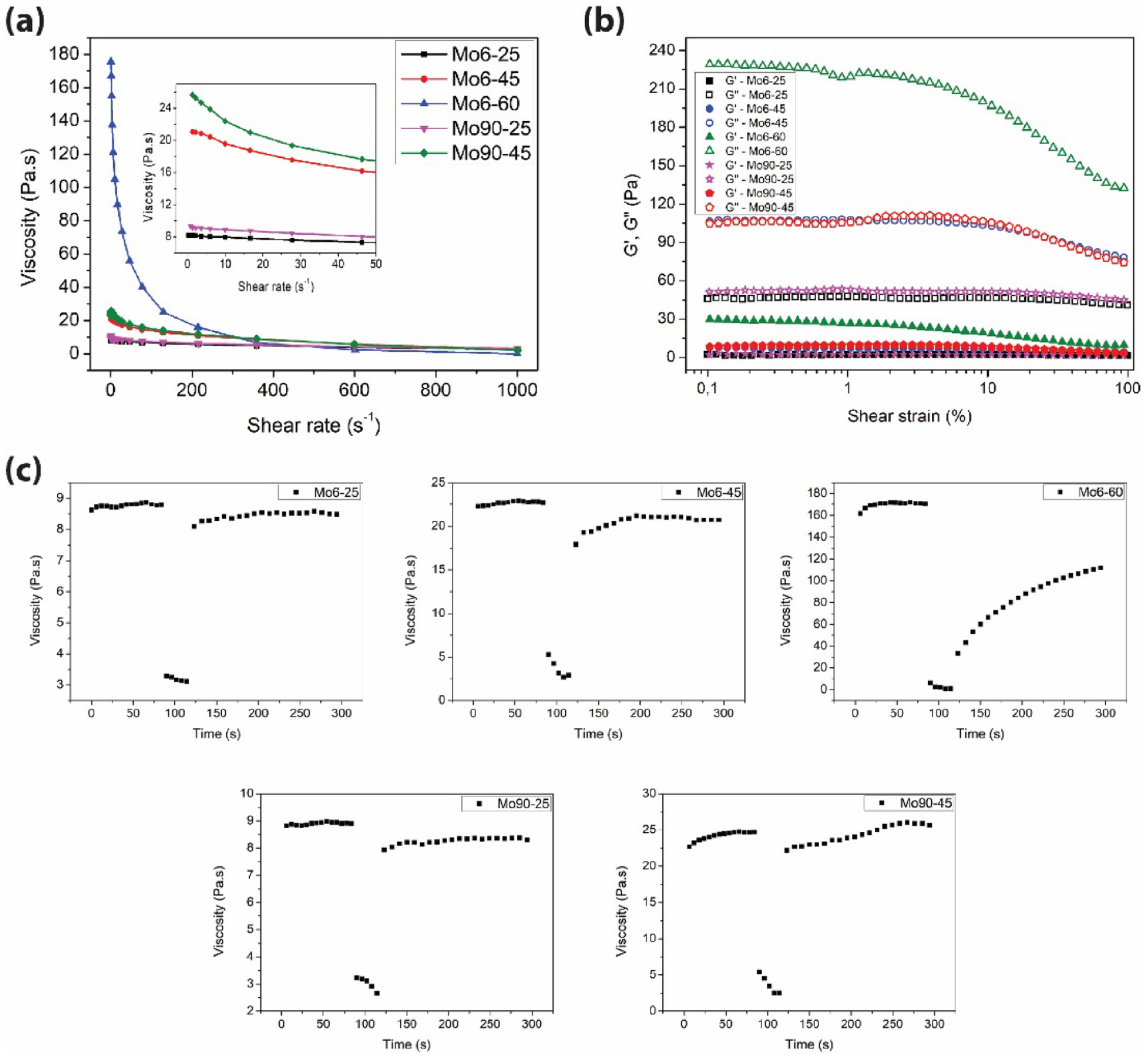
(**a**) Dynamic viscosity of screen-printable MoS_2_ inks at shear rates ranging from 0.01 to 1,000 s^-1^. (**b**) Shear strain sweep of MoS_2_ inks. (**c**) Rheological behavior of MoS_2_ inks during simulation of screen-printing.

In order to investigate the influence of ink formulation on rheological properties, different screen-printable MoS_2_ inks were prepared with various particle sizes and concentrations of MoS_2_. All prepared MoS_2_ inks exhibited typical shear-thinning non-Newtonian rheological behavior (Fig. 1a). It is characterized by a decreasing dynamic viscosity with an increasing shear rate, and it is crucial for the screen-printing process^24^. The increasing shear rate adversely affects the cohesive internal forces between the particles and the binder and destroys the ink structure, which leads to an unsteady ink system^26^. When the shear rate increased from 0.001 to 1000 s^−1^, the dynamic viscosity of all inks dropped gradually. It is obvious that dynamic viscosity increases with MoS_2_ concentration. The viscosity of Mo6-60 sample with the highest MoS_2_ content was slightly higher than what would be optimum for screen-printing. The maximum value was 105 Pa.s at 10 s^−1^, and the minimum value was 0.02 Pa.s at 1000 s^−1^. This rapid decrease indicates that a higher concentration of MoS_2_ causes a faster breakdown of the structure. It is also clear that particle size does not significantly affect dynamic viscosity. Values of dynamic viscosity are identical (the maximum was ~ 9 Pa.s and the minimum was 3 Pa.s) for samples Mo6-25 (particle size ~ 6 µm) and Mo90-25 (particle size ~ 90 nm) containing 25 wt% of MoS_2_. The same values (the maximum was ~ 22 Pa.s and the minimum was ~ 2 Pa.s) were obtained also for samples Mo6-45 (particle size ~ 6 µm) and Mo90-45 (particle size ~ 90 nm) containing 45 wt% of MoS_2_.

The viscosity of screen-printable inks has to be low enough (under applied external force) to allow the squeegee to press the ink through the screen mesh, but also high enough (after releasing the force) to support ink to retain the geometry of printed patterns^24^. The rheological behavior of MoS_2_ inks during the screen-printing process simulated by the time-dependent controlled-shear-rate tests is shown in Fig. 1b, and the characteristic parameters are listed in Supplementary Table S1. Screen-printing process can be divided into three steps. The first interval of the curve simulates the behavior of the ink at rest on the screen at a pre-set low shear rate (0.5 s^−1^ for 90 s). The second interval corresponds to the structural breakdown of the ink when it is pressing through the openings in the screen. The shear rate increases up to 1000 s^−1^ for 30 s. Finally, in the third interval, the shear rate decreases to 0.5 s^−1^ for 120 s to allow structural regeneration evaluation. During the shear rate increase from 0.5 to 1000 s^−1^ in the second interval, the viscosity value drops significantly (Fig. 1b and Supplementary Tab. S1). The applied shear stress destroys the internal structure of the inks. Subsequently, the ink structure is rebuilt and restored when the shear rate returns to 0.5 s^−1^ in the third interval^26^. The viscosity of Mo6-25 and Mo90-25 was the lowest (8.8 and 8.9 Pa.s) at 0.5 s^−1^, and these samples showed the highest recovery rate of 97% and 94% in 250 s, respectively. In addition, the rheological behavior during the process of the screen-printing simulation was the same for samples containing 25 wt% of MoS_2_ (Mo6-25, Mo90-25) and also for the samples containing 45 wt% of MoS_2_ (Mo6-45, Mo90-45). Thus, the particle size does not have a noticeable effect on the printing paste rheology within the printing process. The regeneration of these four samples was quick from the beginning, with the rise of the curve being slow afterwards. The recovery rate of sample Mo90-45 is slightly over 100% in 250 s (Fig. 1b). Thus, if the third interval was longer, viscosity would drop back to (or even under) the reference value^27^. The result of the screen-printing simulation process for the sample Mo6-60 is different from the samples with lower MoS_2_ concentrations. The initial viscosity is too high (170 Pa.s), and as the shear rate increased to 1000 s^−1^, the viscosity decreased to 2.5 Pa.s. In the third interval, the regeneration is slower, indicating a longer time for levelling of the applied layers after printing, resulting in a smoother surface. Despite the recovery rate being only 60%, due to the slower increase in viscosity during the regeneration step, sample Mo6-60 can be considered the most suitable from a layer topography point of view.

To investigate the impact of MoS_2_ concentration and particle size on the viscoelastic properties of the inks, oscillatory measurements were carried out for prepared inks, specifically shear strain amplitude sweep tests. Figure 1c shows the storage (${G}^{\prime}$) and loss modulus (${G}^{\prime\prime}$) dependences of prepared MoS_2_ inks at shear strain varying from 0.1 to 100%. The curve shapes of the samples with the same concentration of MoS_2_ (samples Mo6-25 and Mo90-25; samples Mo6-45 and Mo90-45) are similar even though they had different particle sizes. The oscillatory test results (values of LVR, ${G}^{\prime}$ and ${G}^{\prime\prime}$) for MoS_2_ inks are listed in Table 1.

**Table 1:** The oscillatory strain sweep parameters in the LVR.

Sample	LVR end point (%)	${G}^{\prime}$ at LVR end point (Pa)	${G}^{\prime\prime}$ at LVR end point (Pa)	${G}^{\prime\prime}/{G}^{\prime}$ at LVR end point
Mo6-25	41.11	2.16	43.83	20.29
Mo6-45	10.88	7.56	103.81	13.73
Mo6-60	1.65	28.76	222.37	7.73
Mo90-25	34.73	2.44	51.66	21.17
Mo90-45	9.01	9.59	90.19	9.40

The LVR is defined as the region in which the ink can endure mechanical deformation without destroying the structure^28^. LVR values are determined from the ${G}^{\prime}$ curves. Beyond the LVR, the values of ${G}^{\prime}$ and ${G}^{\prime\prime}$ are continuously decreasing for all samples, indicating the gradual internal structure breakdown. The highest LVR had samples containing 25 wt% of MoS_2_ (< 42%), followed by samples with 45 wt% of MoS_2_ (< 11%), and the lowest LVR had a sample with 60 wt% of MoS_2_ (< 2%). All samples show liquid behavior (${G}^{\prime\prime}$> ${G}^{\prime}$) across the entire strain amplitude range. From Table 1, the ${G}^{\prime}$ increase with MoS_2_ concentration, measured values for Mo6-25, Mo90-25, Mo6-45, Mo90-45 and Mo6-60 are 2.16, 2.44, 7.56, 9.59 and 28.76, respectively. Further investigation of the inks elastic behavior was carried out by analyzing the ratio of liquid-like to solid-like bahavior (${G}^{\prime\prime}/{G}^{\prime}$) , the loss factor—tanδ within the LVR (Table 1). A tanδ < 1 demonstrates that the ink is elastic, cohesive or tacky^26^. All prepared MoS_2_ inks have a loss factor tanδ > 1, which indicates viscous behavior^24^. Decreasing loss factor tanδ within the LVR indicates that the MoS_2_ content can positively impact the inks' structural strength and elasticity^29^.

**Figure 2:**
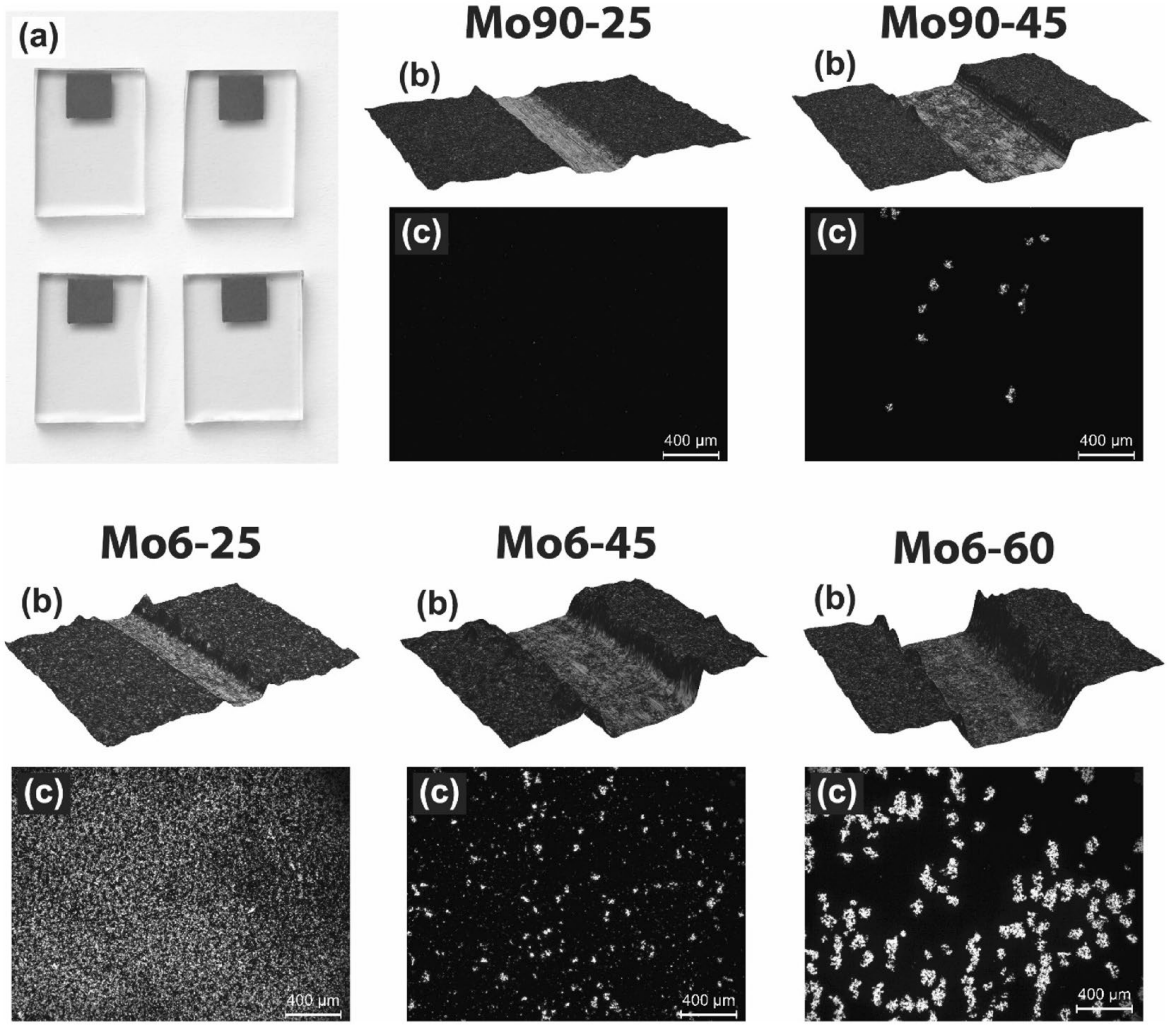
(**a**) Screen-printed MoS_2_ layers onto FTO substrates. (**b**) Needle scratch was made through the middle of the MoS_2_ layers, and the thickness measurement was evaluated by an optical microscope using the 3D image sequential recording method. (**c**) Optical microscopy images of MoS_2_ layers after the peeling test evaluated adhesion to FTO substrates.

After rheology characterization, MoS_2_ inks were screen-printed onto FTO substrates (Fig. 2a). The thickness of MoS_2_ electrodes was evaluated for layers screen-printed onto Al_2_O_3_ ceramic substrates. These substrates were chosen because of their smooth surface, opacity and, from our experience, are more suitable for thin layer thickness measurements. First, a needle scratch was made through the middle of the layers. After that, thicknesses were evaluated by optical microscope using the 3D image sequential recording method (Fig. 2b). As expected, the thickness increased with the concentration of MoS_2_ in the inks, and the measured values ranged from 4 ± 1 to 18 ± 1 µm. Specifically, measured values were 4 ± 1, 11 ± 2 and 18 ± 1 µm for samples Mo6-25, Mo6-45, Mo6-60 and 4 ± 1, 10 ± 1 µm for Mo90-25 and Mo90-45, respectively. As it was seen, particle size did not influence the thickness, but layers based on smaller 90 nm MoS_2_ particles had lower roughness.

Screen-printed MoS_2_ layers have undergone an evaluation of adhesion to FTO substrate by a peeling test. Figure 2c shows optical microscopy images of samples after the peeling test. As it can be seen, layers containing 6 µm MoS_2_ have lower adhesion than layers containing 90 nm MoS_2_. Also, a more significant part of the layer was pulled down with increasing MoS_2_ concentration. Therefore, the highest adhesion to FTO substrate was evaluated for the sample based on 90 nm MoS_2_ with the lowest particle content (Mo90-25). The adhesion to the FTO substrate and the cohesion of the layers are mainly affected by the size of the particle's phase interface, their surface energy, and the concentration of ethylcellulose. Therefore, the degree of adhesion could be increased by finding the ideal ratio between binder, ethylcellulose, and MoS_2_.

**Figure 3:**
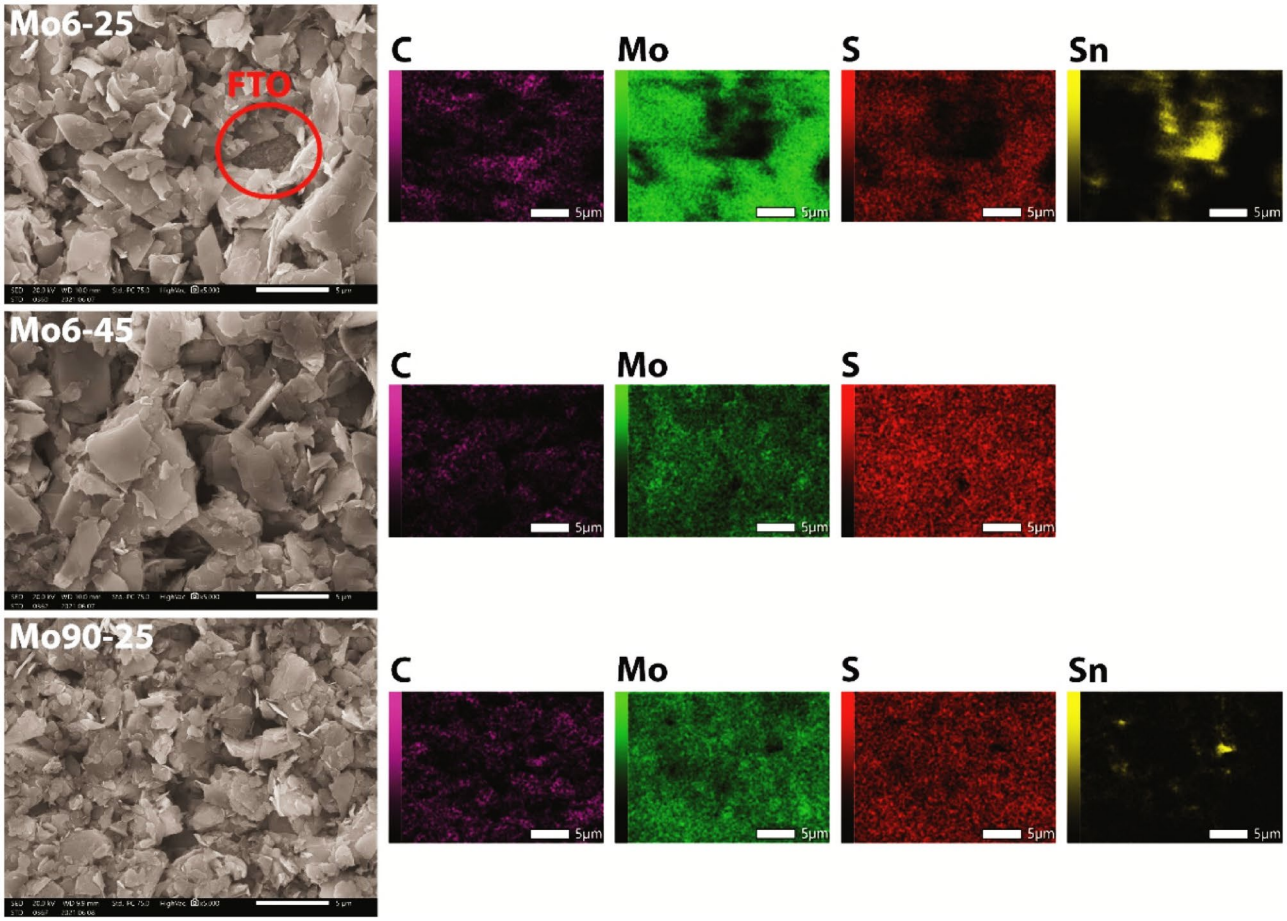
SEM/EDX analysis of printed Mo6-25, Mo6-45 and Mo90-25 layers.

SEM measurements showed (Supplementary Fig. S1) that the particle size in the layers prepared from MoS_2_ particles, characterized by the manufacturer as particles with an average size of 90 nm, actually ranged up to micrometres. Therefore, we assume that the manufacturer's labelling refers more likely to the particles with the highest frequency of occurrence (90 nm) than their average size. Despite the discrepancy, it was evident from the SEM measurements that the average diameter of particles referred to as 90 nm is lower than the diameter of the second particle type used with a more correctly declared average size of 6 µm (Supplementary Fig. S1). In addition, similar to the layer thickness measurements, it is evident that the layers prepared from the 90 nm MoS_2_ inks have a smoother surface, with a higher frequency of submicron particles. Figure 3 shows SEM and EDX mapping results of selected samples Mo6-25, Mo6-45 and Mo90-25. The homogeneity of printed electrodes was insufficient in the case of the lowest MoS_2_ concentrations, regardless of the particle size (Mo6-25 and Mo90-25). As a result, these electrodes did not cover FTO substrates homogenously, as shown in the SEM images or EDX element maps (measured Sn signal from the FTO). Insufficient homogeneity of these MoS_2_ electrodes, related to the measured lowest thickness of 4 µm, can be problematic when applied to electrodes other than FTO. For example, when applied onto Ag electrodes in printed three-electrode systems, which are primarily intended to provide electron transport from the working electrode and can not interact with the analyte solution. Printed electrodes with 45 wt% and 60 wt% MoS_2_ were homogenous and did not expose the surface of FTO substrates. Supplementary Tab. S2 shows the atomic representation of the detected elements, where the carbon intensity corresponds to the added binder ethylcellulose and varies depending on its concentration.

### Electrochemical detection of dopamine by applying screen-printed FTO/MoS_2_ electrodes

**Figure 4:**
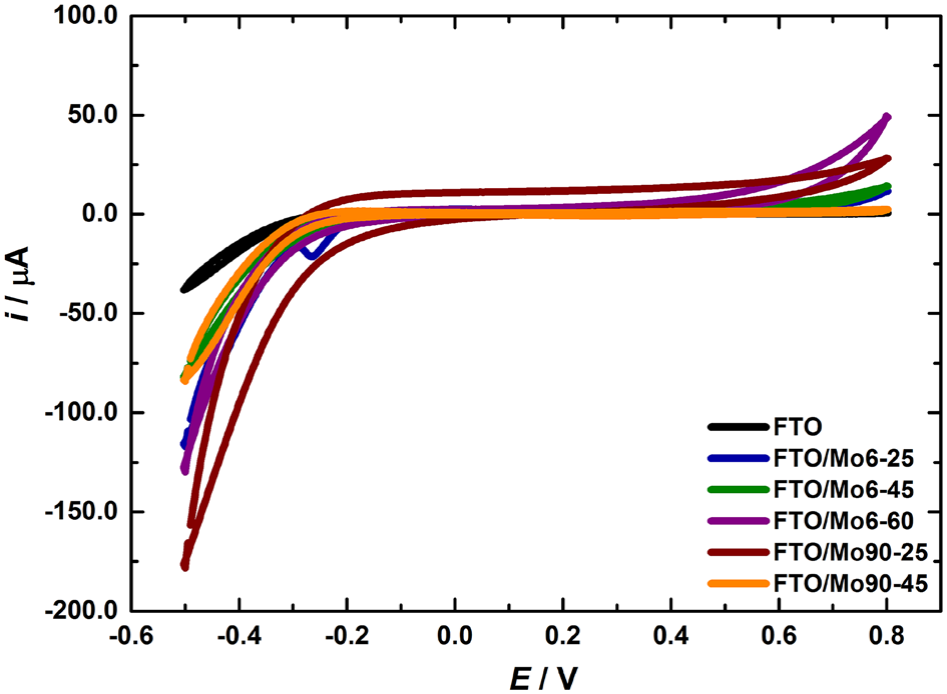
Cyclic voltammograms recorded for screen-printed FTO/MoS_2_ electrodes, *i*.*e*. FTO/Mo6-25, FTO/Mo6-45, FTO/Mo6-60, FTO/Mo90-25 and FTO/Mo90-45, respectively in a potential window of − 0.5 V–0.8 V at a scan rate of 100 mV s^-1^, 20th cycles. Electrolyte: 0.1 M PB pH 7.0.

Electrochemical performance of fabricated FTO/MoS_2_ working electrodes was first evaluated in 0.1 M PB of pH 7.0, employing cyclic voltammetry (CV). Figure 4 shows representative cyclic voltammograms resulting from runs at conductive FTO substrates modified with MoS_2_ in a potential window from − 0.5 V to 0.8 V at a sweep rate of 100 mV s^−1^. Electrochemical investigation of FTO/MoS_2_ electrodes, *i*.*e*. FTO/Mo6-25, FTO/Mo6-45, FTO/Mo6-60, FTO/Mo90-25 and FTO/Mo90-45, in a plain electrolyte revealed that there were no so significant differences in the capacitive current response between screen-printed FTO/MoS_2_ electrodes (Fig. 4).

Dopamine molecule plays an important role in human metabolism, being a crucial neurotransmitter in the central nervous system maintaining neuro-physiological control of mental activities. The concentration of DA ranges between 1 and 2 mM in the intracellular fluids of the central nervous system. Any relevant deviation from an optimal concentration of dopamine in the body causes Parkinson's disease or schizophrenia. Since dopamine is electrochemically active, it may be detected by electrochemical oxidation.

**Figure 5:**
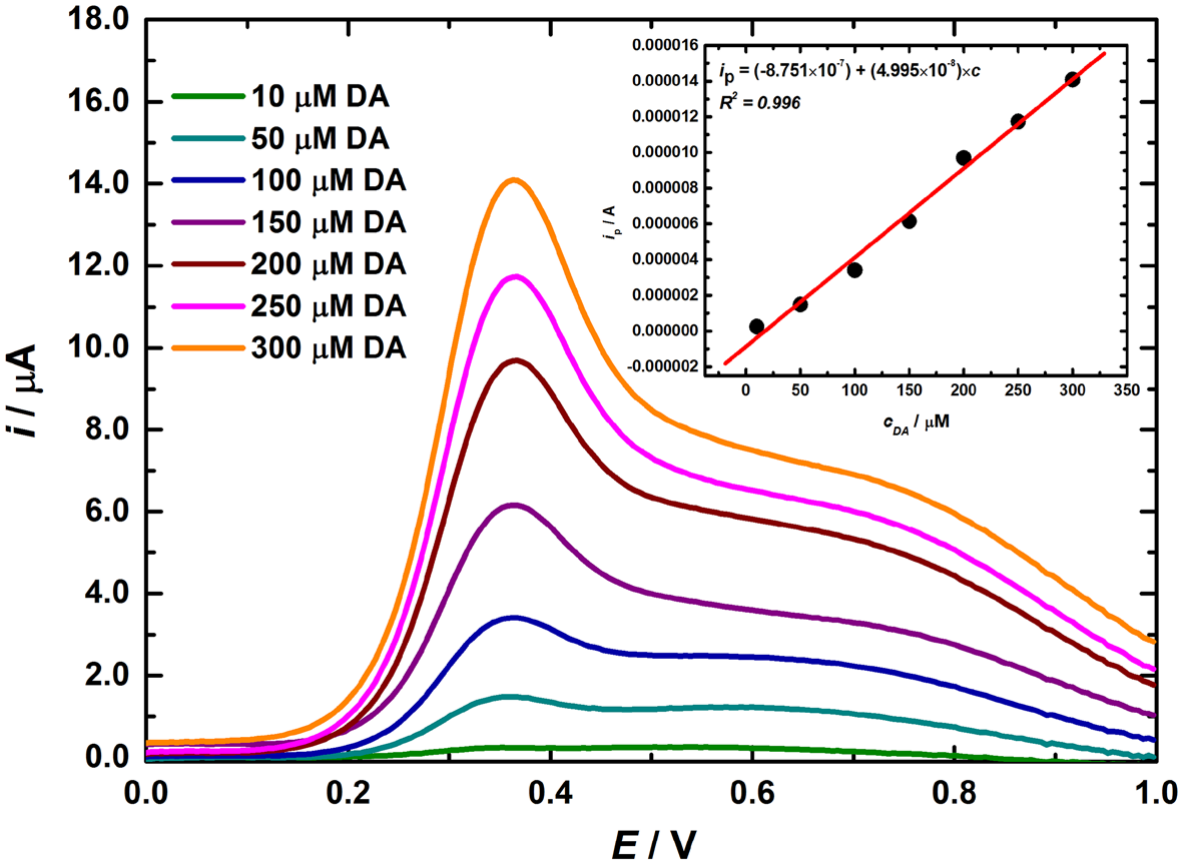
Background-subtracted differential pulse voltammograms obtained for various concentrations of dopamine (DA) at sample FTO/Mo6-45. Electrolyte: 0.1 M PB pH 7.0. Inset figure shows a calibration curve.

The electrochemical behaviour of screen-printed FTO/MoS_2_ electrodes, i.e. FTO/Mo6-25, FTO/Mo6-45, FTO/Mo6-60, FTO/Mo90-25 and FTO/Mo90-45, was assessed by DPV experiment. The DPV curve of sample FTO/Mo6-45 showing electrochemical oxidation of dopamine in 0.1 M phosphate buffer (pH 7.0) as a supporting electrolyte can be clearly seen in Fig. 5. The DPV curves of other samples are shown in Supplementary Fig. S2. The linear dependence of *i*_p_ (oxidation peak current) vs. *c*_DA_ (concentration of dopamine) was investigated in the range up to 300 µM for the measured target analyte. The limit of detection (LOD) was calculated according to S/N = 3. The best LOD value of 246 nM (*R*^2^ = 0.996) was calculated for the Mo6-45 sample with sensitivity of 5.00 × 10^–8^ A μM^−1^. The other modified electrodes exhibited the following LODs: 456 nM (*R*^2^ = 0.982) for Mo90-25, 669 nM (*R*^2^ = 0.975) for Mo6-60, 686 nM (*R*^2^ = 0.995) for Mo6-25 and 865 nM (*R*^2^ = 0.976) for the Mo90-45 sample. The response towards 10 mM DA was 245 nA on an electrode modified by Mo6-45 ink, while a significantly lower response of 45 nA was observed on a bare FTO electrode.

MoS_2_ has been studied as a working electrode material for DA detection in many works by various authors. However, most of this work is devoted to sophisticated MoS_2_ electrodes modified with other materials and prepared by different techniques, which are not as suitable for mass production of working electrodes as printing. Moreover, printing (especially screen-printing) brings several advantages, such as simple, cheap, fast and reproducible preparation of electrodes in large areas on various and flexible substrates. Below, for comparison, are briefly mentioned works of other authors dealing with the preparation of MoS_2_ sensors for DA detection prepared by various methods and based on different, primarily composite MoS_2_ sensing platforms. It is clear from the comparison that the screen-printed MoS_2_ working electrodes achieve not a best but comparable LOD in DA detection.

The individual and simultaneous detection of DA with LOD of 0.58 μM was performed by applying the redox-active dye Methylene Blue (MB) grafted onto MoS_2_ nanosheet surface (MoS_2_@MB) via electrostatic and π-stacking interaction^30^. Velmurugan and Yang obtained LOD of 1.6 nM for DA employing a MoS_2_ − graphitic carbon nitride (MoS_2_ − GCN) p − n heterojunction stabilized reduced graphene oxide/indium tin oxide (rGO/ITO) photoelectrode^13^. Additionally, more papers were previously published describing DA sensors employing graphene or MWCNTs and MoS_2_ composites^13,15,31–34^. Moreover, instead of carbon-based nanomaterials, metallic nanoparticles (Au, Ag, Pt) in combination with MoS_2_ were successfully applied to develop sensitive DA sensors^16,35–39^. Sabar et al. constructed a flexible and economically viable electrochemical sensor for DA detection consisting of the carbon cloth (CC) as a host interface for direct growth of MoS_2_NS via hydrothermal methodology^40^. Golf ball-like MoS_2_ nanosheet arrays (diameter of ∼ 2 μm) deposited on carbon nanofibers (CNFs) proved excellent electrochemical properties for the detection of DA^37^. The MoS_2_ nanosheets incorporated into poly(3,4-ethylenedioxythiophene) (PEDOT) by electrodeposition onto GCE formed a nanocomposite detecting DA with LOD of 0.52 μM^41^. The amperometric biosensor (CP-MoS_2_-R-Nafion:TBAB-Lac) for DA detection was fabricated by the modification of the carbon paper electrode surface with MoS_2_ in the form of ribbons (MoS_2_-R) and flowers (MoS_2_-F), coupled with the immobilization of laccase enzymes^42^. Instead of other types of electrodes, the disposable SPCE were patterned with 2D-MoS_2_ to develop an electrochemical biosensor for detection of DA achieving a sensitivity value of 1044 μA mM^−1^ cm^−2^^17^. Lei et al. fabricated an electrochemical DA sensor based on Mn-doped MoS_2_ synthesized via a scalable two-step approach (with Mn ~ 2.15 at. %)^43^. Zhang with co-workers found out that while MoS_2_ nanosheets catalyzed the electro-oxidation process of Ru(bpy)_3_^2+^ to generate Ru(bpy)_3_^3+^, DA inhibited the effect on electrogenerated chemiluminescence (ECL) intensity of Ru(bpy)_3_^2+^-MoS_2_ nanosheets through the energy transfer process. LOD of 8.5 × 10^−10^ mol L^−1^ was obtained for DA^44^. All mentioned works are summarized in Table 2.

**Table 2:** Comparison of analytical performance of various DA electrochemical sensors depending on sensing platform modification, detection and deposition technique.

Electrode platform	Modification of sensing surface area	Detection technique	LOD obtained for DA determination (μM)	Linear range (μM)	Deposition technique	References
GCE	Self-assembled AuNPs@MoS_2_-NSs	CV	1.0	5.0–200.0	Drop-casting	Zou et al.^35^
GCE	MoS_2_@MB nanohybrid	DPV	0.58	1.0–500.0	Modifying GCE by MoS_2_@MB film	Su et al.^30^
GCE	MoS_2_-RGO BC_5_N	DPV	MoS_2_-Gr/GCE: 0.55 BCN/GCE: 2.1	MoS_2_-RGO: 1–110 BCN/GCE: 2.3–20	Drop-casting	Pramoda et al.^31^
-	3D Ni/NiO/MoS_2_/rGO foam	CV	0.09	0–3	Hydro-thermal method/Electro-deposition	Zhang et al.^32^
CPE	MoS_2_/Au	DPV	76 × 10^−3^	0.5–300	Drop-casting	Chen et al.^36^
rGO/ITO	MoS_2_ − GCN	PEC sensor	1.6 × 10^−3^	0.005 − 1271.93	Drop-casting	Velmurugan and Yang^13^
CC	MoS_2_NS	CV	0.30	250–4000	Growth of MoS_2_ on CC	Sabar et al.^40^
GCE	AuNPs@MoS_2_	DPV	0.05	0.05–30	Drop-casting	Sun et al.^45^
CNFs	MoS_2_-NSBs	DPV	36 × 10^−3^	1–60	Hydro-thermal method	Yue et al.^37^
GCE	3D-f-MoS_2_-rGO	DPV	0.05	0.2–150	Drop-casting	Ma et al.^33^
GCE	3D-MoS_2_/rGO/Au	DPV	0.11; in a mixture: 0.15	0.5–140.5; in a mixture: 0.3–204.3	Drop-casting	Zhao et al.^38^
GCE	MoS_2_/PEDOT	DPV	0.52	1–80	Electro-deposition	Li et al.^41^
GCE	GNS-CNTs/MoS_2_	DPV	50 × 10^−3^	100 × 10^−3^ -100	Drop-casting	Mani et al.^46^
CP	MoS_2_-R /Nafion:TBAB-Lac (*P*. *sanguineus*)	Amp	10 × 10^−3^	0.1–0.5 1–5	Drop-casting	Rubio-Govea et al.^42^
CPE	Ms-atCNTs (p-Aln/Ms-atCNT)	DPV	0.08	0.6–45	Electro-polymerization	Kumar et al.^34^
ITO	Ag/MoS_2_	Amp	0.20	0.2–50	Spin-coating	Shin et al.^16^
SPCE	2D-MoS_2_	LSV	0.09	1–100	Drop-casting	Zribi et al.^17^
GCE	MoS_2_-CPtNPs	DPV	0.11	1–500	Drop-casting	Zhu et al.^39^
ITO	MoS_2_ NFs-rGO	DPV	0.12	5–60	Spray-coating	Guo et al.^15^
PGSs	Mn-doped MoS_2_	DPV	5 × 10^−3^ in 10% serum	5 × 10^−3^ to 5	Drop-casting	Lei et al.^43^
GCE	pGr-MoS_2_	CV	0.01 × 10^−3^	0.00001–10	Drop-casting	Arya Nair et al.^47^
GCE	MoS_2_NS	ECL	8.5 × 10^−4^	1.0 × 10^−3^- 1.0 × 10^−1^	Spreading on the working area	Zhang et al.^44^
**FTO**	**MoS**_**2**_** (6 μm)**	**DPV**	**0.25**	**1–300**	**Screen-printing**	**This work**

*Ag/MoS*_*2*_ Silver encapsulated MoS_2_ hybrid nanoparticle, *Amp.* Amperometry, *AuNPs* Gold nanoparticles, *BC*_*5*_*N* Borocarbonitride, *CC* Carbon cloth, *CV* Cyclic voltammetry, *CPE* Carbon paste electrode, *CP* Carbon paper, *DA* Dopamine, *ECL* Electrogenerated chemiluminescence, *GCE* Glassy carbon electrode, *GNS-CNTs/MoS*_*2*_ Molybdenum sulfide flowers placed on graphene nanosheets and multiwalled carbon nanotubes, *ITO* Indium tin oxide, *Lac (P. sanguineus)* Laccase isoforms (LacI and LacII) from a native strain of the white-rot fungi known as *Pycnoporus sanguineus*, CS43, *LSV* Linear sweep voltammetry, *MB* Methylene blue, *MoS*_*2*_*-NSs* MoS_2_ nanosheets, *MoS*_*2*_* − GCN* A molybdenum disulfide–graphitic carbon nitride, *MoS*_*2*_*-NSBs* Molybdenum disulfide nanosheets resembling the shape of golf balls, *MoS*_*2*_*-R* Molybdenum disulfide ribbons, *Ms-atCNTs* MoS_2_/acid-treated MWCNTs composite, *MoS*_*2*_*-CPtNPs* Clover-like platinum nanoparticle-supported MoS_2_, *MoS*_*2*_* NFs-rGO* MoS_2_ nanoflowers-reduced graphene oxide, *p-Aln* Polymerized alanine, *pGr* Pulverized graphite, *pGr-MoS*_*2*_ A graphene-molybdenum disulphide nanocomposite, *PEC* Photoelectrochemical sensor, *PGSs* Pyrolytic graphite sheets, *RGO* Reduced graphene oxide, *rGO/ITO* Reduced graphene oxide/indium tin oxide, *SPCE* Screen-printed carbon electrode, *TBAB* Tetrabutylammonium bromide, *3D-f-MoS*_*2*_*-rGO* Worm-like and flower-like molybdenum disulfide (MoS_2_) grown on reduced graphene oxide (rGO), *3D-MoS*_*2*_*/rGO/Au* 3D-networked nanostructure composed of MoS_2_, reduced graphene oxide and gold nanoparticles.

## Conclusion

The screen-printed FTO/MoS_2_ working electrodes for the electrochemical detection of dopamine were successfully prepared in this work for the first time. Screen-printable MoS_2_ high viscosity inks contained MoS_2_ with different particle sizes and concentrations, ethylcellulose and terpineol. The screen-printing simulation confirmed that MoS_2_ inks are suitable for screen-printing process with corresponding flow behavior, and the viscosity recovery rate. Oscillatory modulus determined the linear viscoelastic region and helped to understand the solid- and liquid-like behavior of prepared MoS_2_ inks during screen-printing. After rheological behavior analysis, MoS_2_ inks were screen-printed onto FTO substrates. The differential pulse voltammetry measurements showed that the best limit of detection of 246 nM for DA (S/N = 3) was evaluated for the working electrode printed using the ink containing 45 wt% MoS_2_ powder with an average particle size of 6 μm. Presented results showed that screen-printable MoS_2_ inks could be prepared by a simple, time-efficient mixing process taking only a few minutes. Moreover, MoS_2_ inks prepared in this way allow the fabrication of working electrodes for detection of dopamine or further target analytes by simple, low cost and for mass suitable screen-printing technique.

## Supplementary Information

Supplementary Information.

## Data Availability

All data generated or analyzed during this study are included in this published article.
