# Supplementary material for: Facile fabrication of screen-printed MoS2 electrodes for electrochemical sensing of dopamine
Source: Sci Rep. 2022 Jul 13;12:11900. doi: 10.1038/s41598-022-16187-2 (PMC9277599; doi:10.1038/s41598-022-16187-2)
Supplement: Supplementary file 1 — Supplementary Information. [file 41598_2022_16187_MOESM1_ESM.docx]

**Supplementary Information**

**Facile fabrication of screen-printed MoS_2_ electrodes**

**for electrochemical sensing of dopamine**

Michaela Pavličková^1^, Lenka Lorencová^2^, Michal Hatala^1^,

Miroslav Kováč^1^, Ján Tkáč^2^, Pavol Gemeiner^1*^

^1^Department of Graphic Arts Technology and Applied Photochemistry, Faculty of Chemical and Food Technology, Slovak University of Technology in Bratislava, Radlinského 9,
 812 37 Bratislava, Slovak Republic

^2^Institute of Chemistry, Slovak Academy of Sciences, Dúbravská cesta 9, 845 38 Bratislava, Slovak Republic

**Corresponding author:* [*pavol.gemeiner@stuba.sk*](mailto:pavol.gemeiner@stuba.sk)

**Supplementary Table S1** The viscosity (Pa.s) of MoS_2_ inks at different shear rates during simulation of screen-printing process

|  | **0.5 s^-1^** | **1000 s^-1^** | **0.5 s^-1^**  **at 150 s** | **0.5 s^-1^**  **at 250 s** | **Recovery % at 250 s** |
| --- | --- | --- | --- | --- | --- |
| **Mo6-25** | 8.8 | 3.2 | 8.3 | 8.5 | 97 |
| **Mo6-45** | 22.7 | 3.7 | 19.8 | 21.0 | 93 |
| **Mo6-60** | 170.0 | 2.5 | 60.2 | 102.8 | 60 |
| **Mo90-25** | 8.9 | 3.0 | 8.2 | 8.4 | 94 |
| **Mo90-45** | 24.2 | 3.7 | 23.0 | 25.7 | 103 |


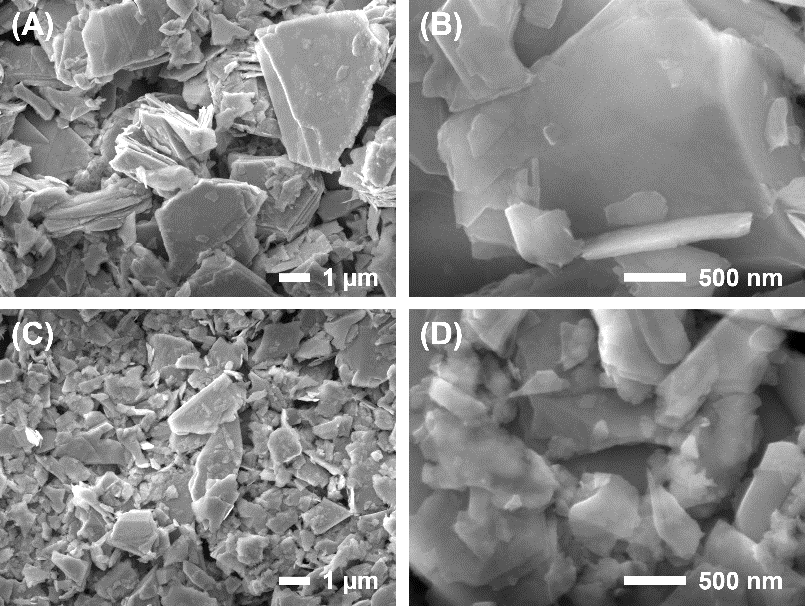


**Supplementary Figure S1** SEM images of screen-printed MoS_2_ electrodes based on particles with average size of ~ 6 µm (A, B) and ~ 90 nm (C,D)

**Supplementary Table S2** Atomic representation of elements in MoS_2_ printed layers obtained by EDX analysis

|  | Atom% | | | | |
| --- | --- | --- | --- | --- | --- |
|  | Mo6-25 | Mo6-45 | Mo6-60 | Mo90-25 | Mo90-45 |
| C | 48.21 ± 0.06 | 41.35 ± 0.06 | 39.55 ± 0.06 | 43.29 ± 0.06 | 41.59 ± 0.06 |
| S | 33.07 ± 0.02 | 41.53 ± 0.02 | 42.75 ± 0.02 | 39.52 ± 0.02 | 41.36 ± 0.02 |
| Mo | 14.02 ± 0.02 | 17.13 ± 0.03 | 17.70 ± 0.02 | 16.41 ± 0.02 | 17.05 ± 0.02 |
| Sn | 4.70 ± 0.01 | - | - | 0.78 ± 0.00 | - |


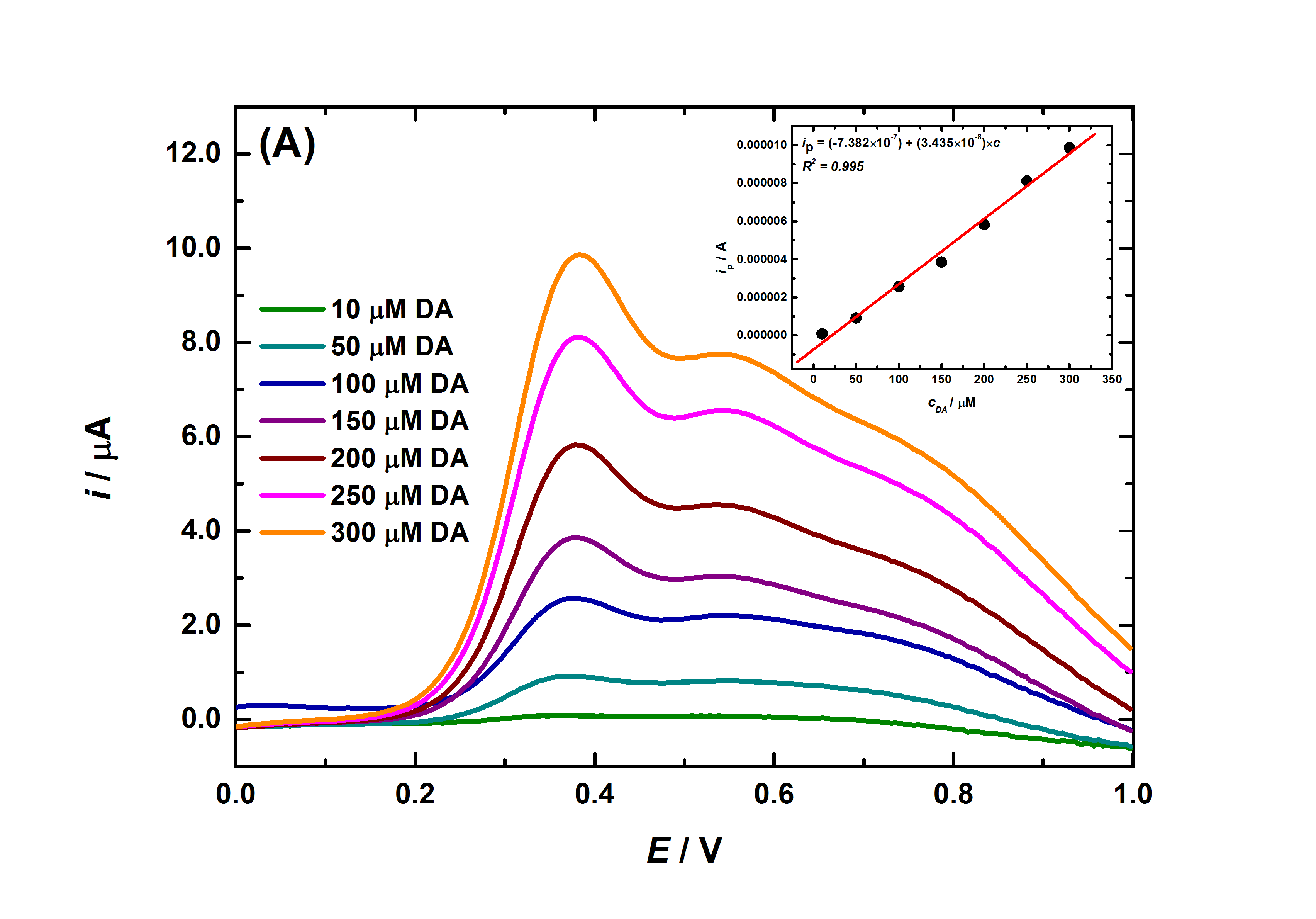

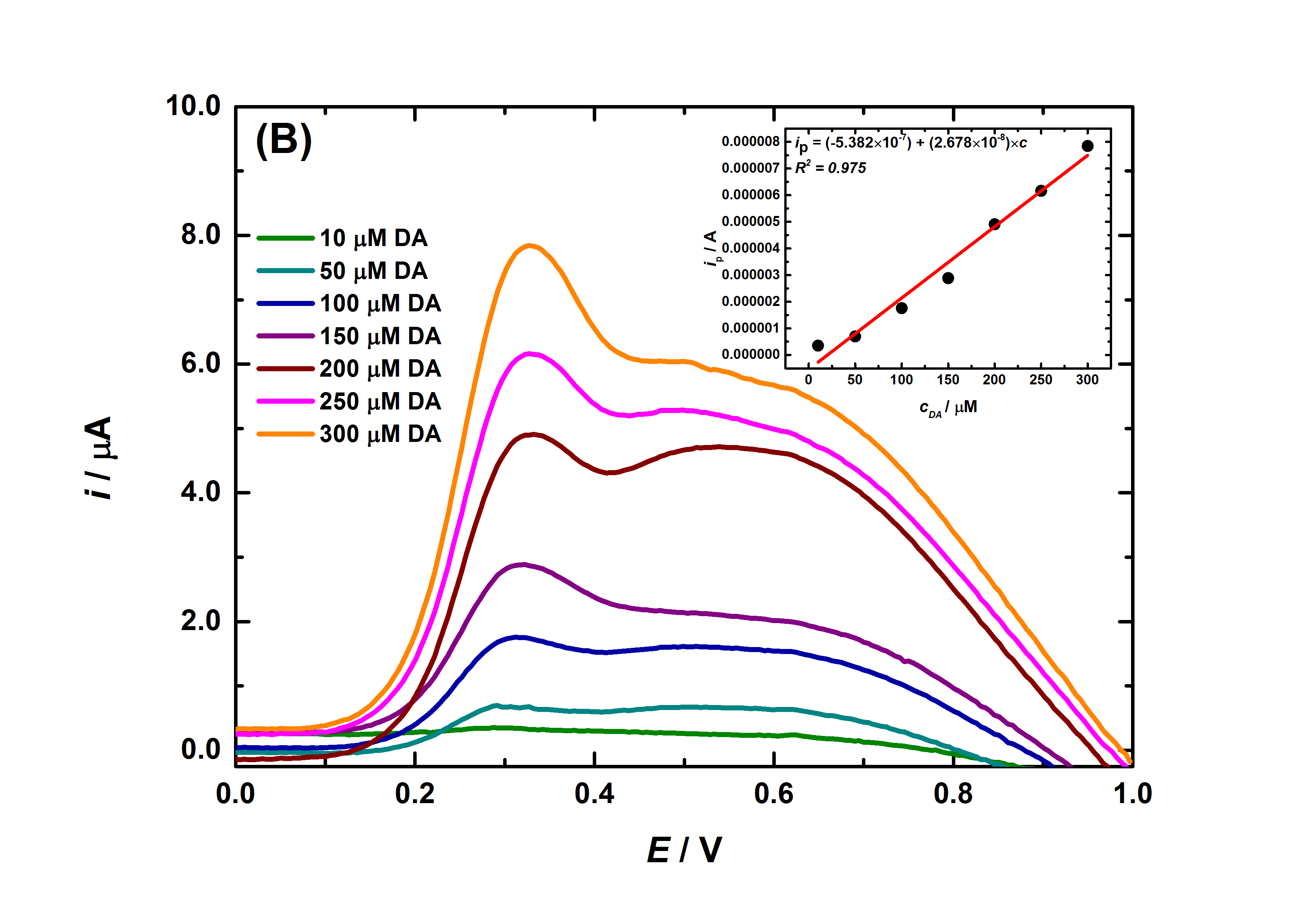


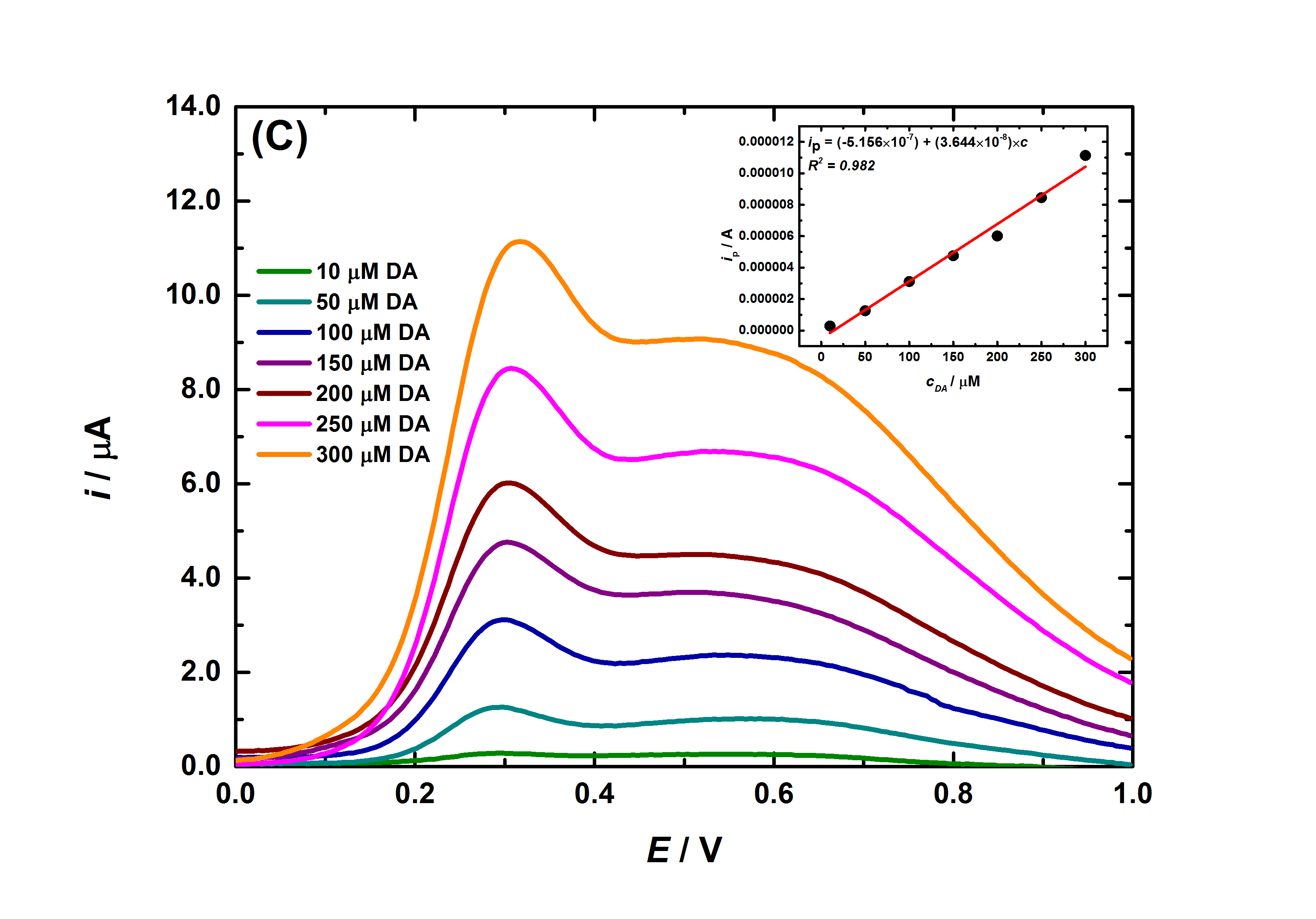

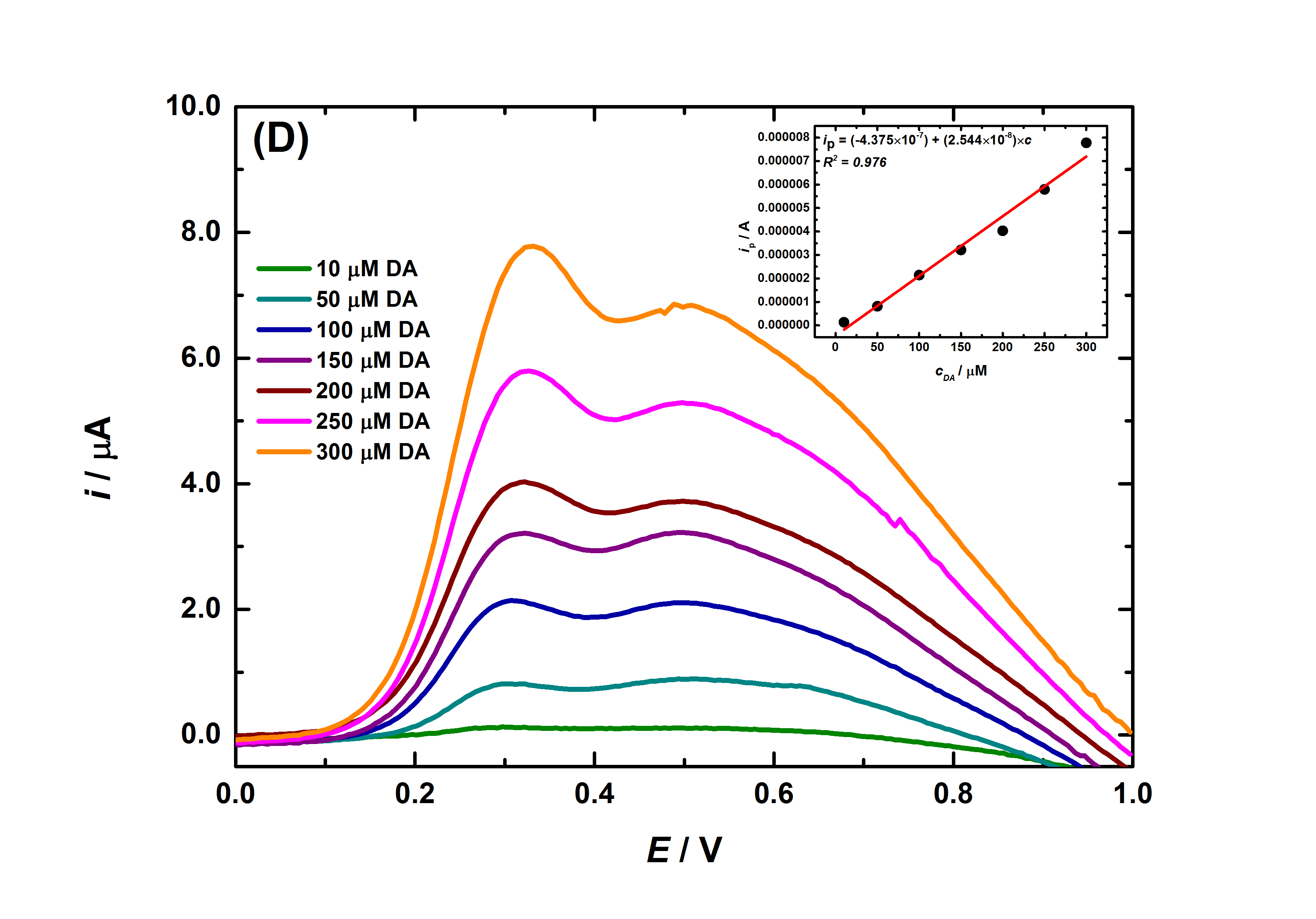


**Supplementary Figure S2** Background-subtracted differential pulse voltammograms obtained for various concentrations dopamine (DA) at (A) FTO/Mo6-25; (B) FTO/Mo6-60; (C) FTO/Mo90-25 and (D) FTO/Mo90-45 electrodes. Electrolyte: 0.1 M PB pH 7.0.
